# Feasibility of totally leadless left bundle branch area pacing via a left endocardial pacing electrode in patients with heart failure: A 3-case series

**DOI:** 10.1016/j.hrcr.2025.07.029

**Published:** 2025-08-07

**Authors:** Elodie Deschamps, Yann Lacombe, Sandrine Venier, Adrien Carabelli, Peggy Jacon, Pascal Defaye

**Affiliations:** Department of Cardiology, University Hospital of Grenoble, Grenoble, France

**Keywords:** Leadless pacing, Conduction system pacing, Left bundle branch area pacing (LBBAP), WiSE-CRT system, Totally leadless CRT, Heart failure, Cardiac resynchronization therapy (CRT), Leadless pacemaker, Micra, Endocardial LV pacing

**Key Teaching Points**

• Left bundle branch area pacing (LBBAP) can be achieved from the left ventricular (LV) septal endocardium using the WiSE-CRT system in patients with heart failure.
• Confirmation of LBBAP requires LV-only pacing to measure LV activation time and assess QRS morphology; however, LV-only mode may not always be maintained during testing.
• Combining the WiSE-CRT system with a Micra device enables a completely leadless cardiac resynchronization therapy approach in selected high-risk patients.
• Despite promising results, challenges remain with device programming and procedural complexity, highlighting the importance of careful patient selection and procedural planning.

## Introduction

**Figure 1:**
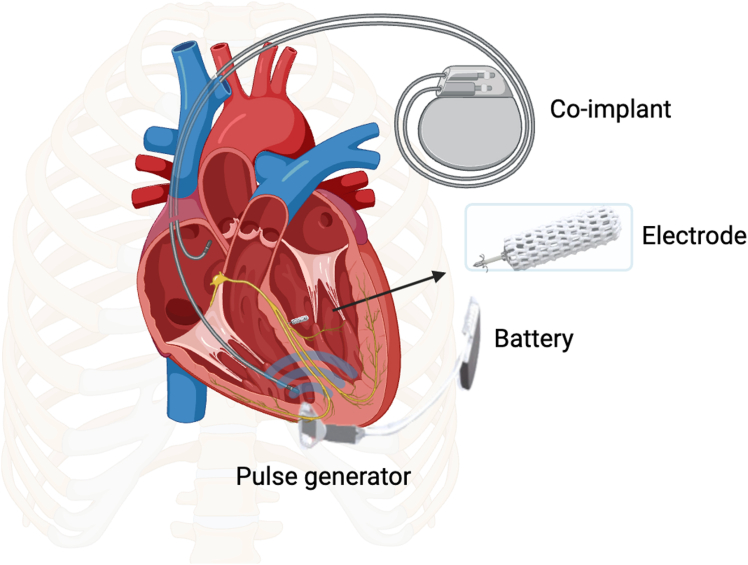
Components of the WiSE CRT system: battery, an ultrasound transmitter, and a receiver, and is combined with a pre-existing RV pacing device. CRT *=* cardiac resynchronization therapy; RV = right ventricle.

Cardiac resynchronization therapy (CRT) is a well-established treatment for heart failure with ventricular dyssynchrony. It is associated with decreased hospitalizations and mortality rates.^1^^,^^2^ However, conventional CRT is not always feasible due to various anatomical constraints, such as coronary sinus cannulation failure, lack of collateral access, or phrenic nerve stimulation. Moreover, 30%–40% of patients are considered non-responders to this therapy according to several studies.^3^, ^4^, ^5^, ^6^ Invasive surgical epicardial lead implantation is rarely proposed in this high-risk population. Left ventricular (LV) endocardial pacing has been explored as an alternative, offering more physiological activation from the endocardium to the epicardium.^7^^,^^8^ Despite these promising results, this approach has been associated with thromboembolic events, infections, and mitral valve dysfunction and has not been adopted.^9^ The WiSE-CRT System (EBR Systems) was developed to address these challenges, enabling leadless LV endocardial pacing via ultrasound energy.^10^ Figure 1 shows the components of the WiSE-CRT. Preclinical studies demonstrated full endothelialization within 30 days post-implant.^11^ Observational studies confirmed both efficacy and safety, and the multicenter stimulation of the left ventricular endocardium for cardiac resynchronization therapy) study met criteria at 6 months with a complication rate similar to other conventional CRT studies.^12^^,^^13^ Based on this, current guidelines support WiSE-CRT as a second-line therapy when conventional CRT fails.^14^ Recently, conduction system pacing, in particular left bundle branch area pacing (LBBAP), has emerged as a physiological alternative to biventricular pacing (BiVP).^15^^,^^16^ LBBAP has shown outcomes comparable to CRT, with larger randomized trials expected to confirm these findings.^17^^,^^18^ Elliot et al^19^ first described the deployment of the WiSE-CRT endocardial electrode in the LV septum, demonstrating the feasibility of LBBAP. In this case series, we report our center’s experience in LBBAP with the WiSE-CRT.

### Case 1: WiSE in combination with CRT-D

A 61-year-old man with nonischemic cardiomyopathy (NICM) and LV ejection fraction (LVEF) 35% had a prior CRT-defibrillator (CRT-D) implanted for primary prevention. Several years later, the LV lead was dysfunctional (high threshold, phrenic stimulation). He was referred for WiSE-CRT as an alternative. The WiSE-CRT system was implanted in a 2-step process as previously reported.^10^^,^^20^ A transthoracic echocardiography (TTE) was performed to identify the optimal intercostal space for the transmitter location which provides an acoustic window to the posterior LV that is not impeded by lung tissue (usually between the 4th and 6th intercostal spaces).

**Figure 2:**
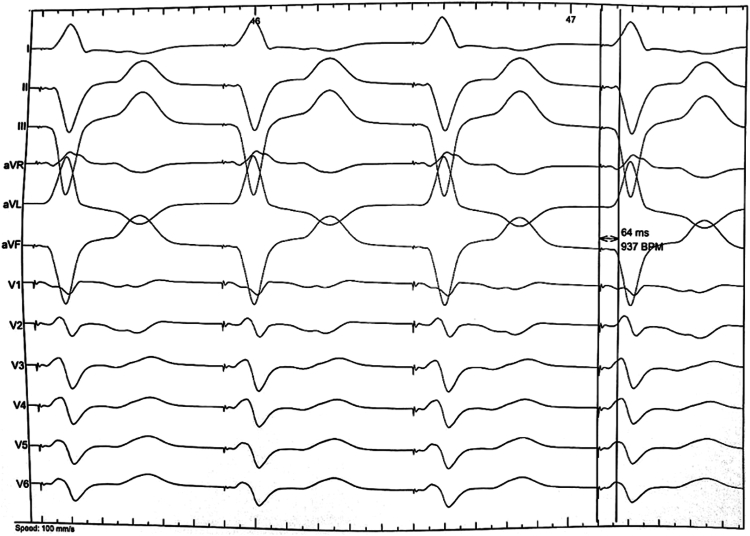
12-lead ECG showing measurement of V6RWPT = 64 ms and small R-wave in lead V1, suggestive of left bundle branch area pacing (LBBAP). ECG = electrocardiogram; RWPT = R wave peak time.

**Figure 3:**
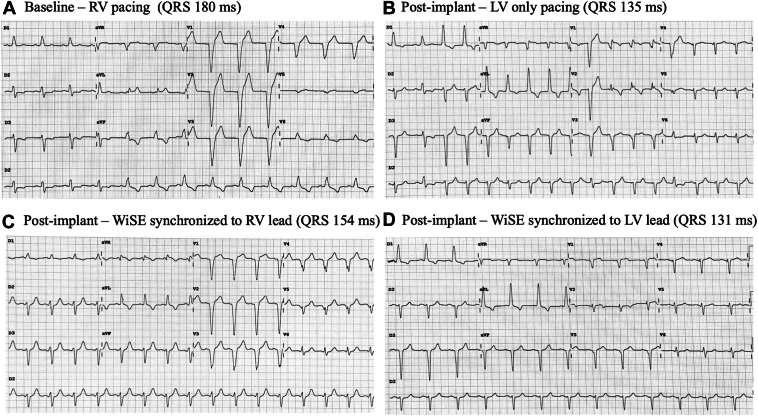
12-lead ECG panel demonstrating QRS morphology at different pacing conditions. ECG = electrocardiogram; RV = right ventricle; LV = left ventricle.

Stage 1 of the procedure involved implantation of the transmitter in the intercostal space below the pectoralis muscle and battery in the left mid-axillary intermuscular pocket. Two days later, he was readmitted to the operating room for placement of the LV pacing electrode. A Flexcath Advance Stearable sheath (Medtronic) was introduced through the femoral vein into the LV via a transseptal puncture guided by transesophageal echography. An electrophysiology (EP) catheter (Biosense Webster) was used for selection of the optimal site. Potential septal pacing sites were selected under fluoroscopic and echocardiographic guidance, using QRS morphology and stimulus-to-QRS latency as indicators. Once an appropriate septal endocardial LV pacing site was identified, the tiny passive electrode was deployed and anchored into the LV endocardium. Supplemental Video 1 provides an example of fluoroscopic deployment of the WiSE-CRT electrode on the LV septum. Final position was mid-septum. LV-only pacing met LBBAP criteria with right bundle branch block pattern and V6 R wave peak time (RWPT) at 64 ms (Figure 2). Additionally, LV-only pacing produced shorter QRS compared with BiVP and right ventricular (RV)-only pacing (Figure 3). At 6-month follow-up, the patient reported a reduction in symptoms with New York Heart Association class II and 100% BiVP. TTE revealed an increase in LVEF to 41.5% and reduction in LV end-diastolic volume (LVEDV).

### Case 2: WiSE in combination with Micra VR pacemaker

**Figure 4:**
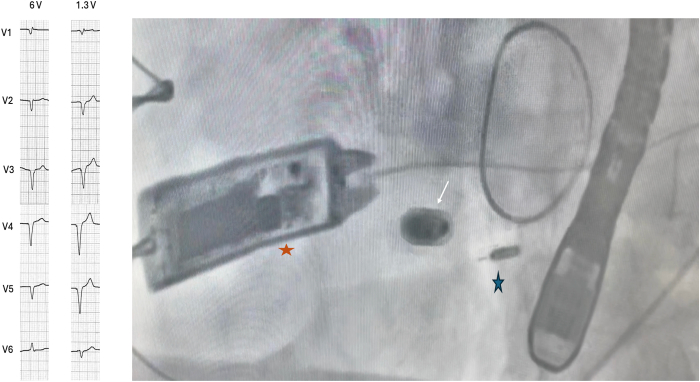
Left*:* QRS morphology changes at high (6 V) and low (1.3 V) output. Right*:* Fluoroscopic view after implantation showing LV electrode (blue star), transmitter (orange star), and Micra VR (white arrow). LAO = left anterior oblique; LV = left ventricle.

**Figure 5:**
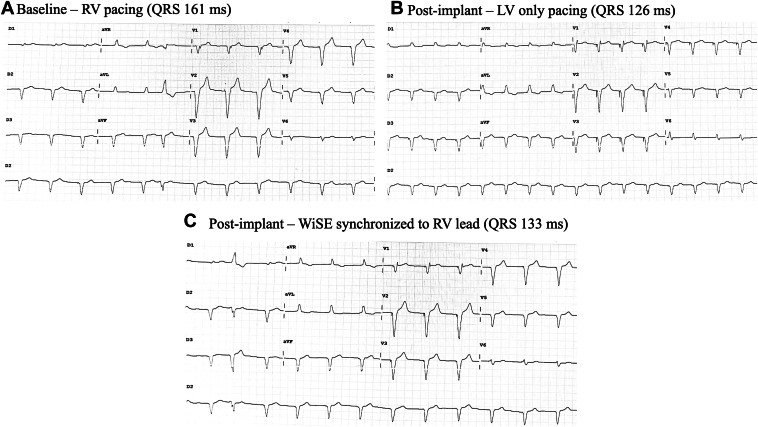
12-lead ECG and fluoroscopic panel. **A:** Baseline RV pacing, **B:** LV only pacing, **C:** WiSE-CRT system synchronized to RV pacing. CRT, cardiac resynchronization therapy; ECG = electrocardiogram; LV = left ventricle; RV = right ventricle.

An 81-year-old man known for mitral valvuloplasty had a prior Micra VR (Medtronic, Minneapolis, MN) implanted for atrioventricular block due to tricuspid insufficiency. He then developed pacemaker-induced cardiomyopathy (PICM) with decreased LVEF at 35%. A WiSE-CRT system upgrade was preferred to preserve the leadless pacemaker. The WiSE-CRT system was implanted following the previous steps. The electrode was finally deployed in the mid-inferoseptum with a V6RWPT at 58 ms. During testing, a discrete QRS transition was observed, with lower output (1.3V) revealing narrower QRS and the appearance of an S wave in lead V6 (Figure 4). LV-only pacing successfully showed greater electrical resynchronization (QRS duration 126 ms) compared with BiVP (QRS 133 ms) and baseline RV pacing (161 ms) (Figure 5). At 6-month follow-up, the WiSE-CRT system achieved 99% BiVP; the patient reported an improvement in quality of life, and echocardiography showed an increase in LVEF to 46%.

### Case 3: WiSE in combination with Micra AV pacemaker

**Figure 6:**
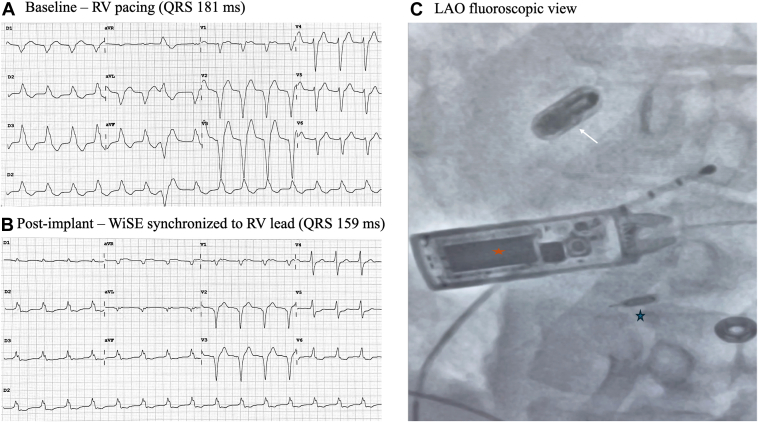
Fluoroscopic and ECG composite panel. **A**: RV pacing, **B:** Biventricular pacing using WiSE-CRT synchronized to RV lead, and **C:** Final fluoroscopic image showing the implanted WiSE-CRT system components. CRT, cardiac resynchronization therapy; ECG = electrocardiogram; RV = right ventricle.

A 60-year-old man with NICM and reduced LVEF of 33% was implanted a few years ago with a CRT-D for primary prevention. He also had poorly controlled diabetes and peripheral artery disease with a prior left leg amputation. He developed infective endocarditis requiring device and lead extraction, and a totally leadless CRT system was preferred. A Micra AV (Medtronic, Minneapolis, MN) was implanted as a first step. The WiSE-CRT system was initially positioned in the basal anteroseptal segment, but showed a high capture threshold. The device was finally relocated to a basal inferoseptal position (Figure 6). However, the system could not be programmed in LV-only pacing mode owing to device constraints, preventing measurement of the V6RWPT and the acquisition of a 12-lead electrocardiogram (ECG) under this configuration. Based on the anatomical position and QRS narrowing during BiVP, at least LV septal pacing was assumed. A stable LV capture threshold and a high BiVP percentage based on device interrogation was reported at discharge. Unfortunately, the patient died 1 month later due to terminal heart failure associated with cardiorenal syndrome.

A summary of clinical, electrical, and echocardiographic parameters before and after WiSE-CRT implantation in the presented cases is shown in Table 1.

**Table 1:** Clinical, electrical, and echocardiographic characteristics of the 3 patients

Parameters	Case 1	Case 2	Case 3
Age (years)	61	81	60
Cardiomyopathy	NICM	PICM	NICM
Co-implant	CRT-D	Micra VR	Micra AV
LV electrode location	Mid-septal	Mid-inferoseptal	Basal-inferoseptal
Transmitter-electrode distance (cm)	7.3	6.3	8
Angle (°)	-31	18	12
LV pacing threshold (V @ ms)	1 V @ 0.15	1 V @ 0.3	1 V @ 0.05
Biventricular pacing at 6 months (%)	100	99	NA
QRS duration (ms) – baseline	170∗	161∗	181∗
QRS duration (ms) – post-implant	154	133	159
LVEF (%) – baseline	35	35	33
LVEF (%) – at 6 months	41.5	46	NA
LVESV (mL) – baseline	140	96	172
LVESV (mL) – at 6 months	78	62	NA
LVEDV (mL) – baseline	213	148	260
LVEDV (mL) – at 6 months	134	118	NA
NYHA – baseline	III	II	III
NYHA – at 6 months	II	II	NA

LV = left ventricular; LVEF = left ventricular ejection fraction; LVEDV = left ventricular end-diastolic volume; LVESV = left ventricular end-systolic volume; NA = not applicable; NICM = non-ischemic cardiomyopathy; NYHA = New York Heart Association; PICM = pacemaker induced cardiomyopathy; CRT-D = cardiac resynchronization therapy defibrillator.

∗ RV pacing.

## Discussion

This case series demonstrated the feasibility of LBBAP via the LV septal approach using the WiSE-CRT system. LBBAP has emerged as a promising alternative to BVP, with growing evidence of superiority in QRS narrowing, LVEF, and clinical outcomes.^17^^,^^18^^,^^21^ However, LBBAP is associated with a risk of septal perforation, embolic events,^22^ and acute failure rate up to 18% in CRT candidates.^23^ Leadless pacing may overcome these limitations. WiSE-CRT was initially designed for lateral LV pacing, but septal delivery has been reported.^19^ In our experience, mapping was performed using a standard EP catheter under fluoroscopic guidance. Pacing morphology and stimulus-to-QRS latency were used to identify an appropriate septal site. No left bundle potential was recorded, and no high-resolution electroanatomical mapping was available. LBBAP criteria, based on QRS morphology and V6RWPT, were met in 2 out of 3 cases. In case 3, since the system could not operate in LV-only mode, neither V6RWPT nor isolated ECG could be obtained, precluding definitive confirmation of LBBAP. However, it would have been technically feasible to assess LV-only QRS morphology and RWPT (by reducing RV output below threshold, allowing the LV electrode to trigger on the pacing artifact. This approach should be considered in future procedures. Although combinations of Micra and WiSE-CRT have been previously described to achieve totally leadless CRT, these cases used lateral wall LV pacing. To our knowledge, this may be the first reported case series of totally leadless CRT combining WiSE-CRT-guided LBBAP with Micra. This approach is particularly attractive for patients with prior infections, vascular access issues, or comorbidities.

## Limitations

Several limitations and uncertainties must be highlighted. First, the long-term outcomes of this approach remain unknown, and it is essential to confirm the long-term electrical stability of the electrodes, sustained clinical efficacy, and absence of late complications. In addition, although LBBAP criteria seem to be met during LV-only pacing, the clinical pacing provided by the WiSE-CRT system is synchronized with RV pacing, resulting in a QRS morphology corresponding to a fusion between LBBAP and RV pacing. Thus, these results should be interpreted with caution. Also, the potential benefits of LBBAP compared with traditional LV endocardial pacing have not yet been studied. Importantly, the cost of the WiSE-CRT system and the requirement for a staged procedure might present substantial barriers to broader adoption of this technology. The percentage of BiVP was assessed through device interrogation, no ambulatory monitoring was performed. Lastly, although the reported complication rate is comparable to conventional CRT methods, specific risks associated with the completely leadless approach (electrode migration, loss of capture, procedural risks related to transseptal puncture, and complications from multiple leadless device implants) should not be overlooked and require careful monitoring. Prospective randomized clinical studies will clarify the role of WiSE-CRT combined with LBBAP in the therapeutic arsenal for refractory heart failure.

## Conclusion

In conclusion, WiSE-CRT is a feasible technique for leadless LBBAP. In addition, totally leadless CRT with LBBAP via the WiSE-CRT system in combination with Micra appears to be a good alternative to conventional CRT in patients with heart failure.

## Disclosures

The authors have no conflicts of interest to disclose.

## Appendix Supplementary Data

Supplemental Material

Video1

## References

[1] Gold M.R., Daubert C., Abraham W.T. (2015). The effect of reverse remodeling on long-term survival in mildly symptomatic patients with heart failure receiving cardiac resynchronization therapy: results of the REVERSE study. Heart Rhythm.

[2] Abraham W.T., Fisher W.G., Smith A.L. (2002). Cardiac resynchronization in chronic heart failure. N Engl J Med.

[3] Moss A.J., Hall W.J., Cannom D.S. (2009). Cardiac-resynchronization therapy for the prevention of heart-failure events. N Engl J Med.

[4] Bristow M.R., Saxon L.A., Boehmer J. (2004). Cardiac-resynchronization therapy with or without an implantable defibrillator in advanced chronic heart failure. N Engl J Med.

[5] Bleeker G.B., Bax J.J., Fung J.W.H. (2006). Clinical versus echocardiographic parameters to assess response to cardiac resynchronization therapy. Am J Cardiol.

[6] van Rees J.B., de Bie M.K., Thijssen J., Borleffs C.J.W., Schalij M.J., van Erven L. (2011). Implantation-related complications of implantable cardioverter-defibrillators and cardiac resynchronization therapy devices. J Am Coll Cardiol.

[7] Padeletti L., Pieragnoli P., Ricciardi G. (2012). Acute hemodynamic effect of left ventricular endocardial pacing in cardiac resynchronization therapy. Circ Arrhythm Electrophysiol.

[8] Spragg D.D., Dong J., Fetics B.J. (2010). Optimal left ventricular endocardial pacing sites for cardiac resynchronization therapy in patients with ischemic cardiomyopathy. J Am Coll Cardiol.

[9] Rademakers L.M., van Gelder B.M., Scheffer M.G., Bracke F.A. (2014). Mid-term follow up of thromboembolic complications in left ventricular endocardial cardiac resynchronization therapy. Heart Rhythm.

[10] Reddy V.Y., Miller M.A., Neuzil P. (2017). Cardiac resynchronization therapy with wireless left ventricular endocardial pacing. J Am Coll Cardiol.

[11] Echt D.S., Moore D., Cowan M., Valli V.E., Whitehair J.G., Willis N.P. (2010). Chronic im- plantation of leadless pacing electrodes in the left ventricle of a goat model. Heart Rhythm.

[12] Singh J.P., Abraham W.T., Auricchio A. (2019). Design and rationale for the stimulation of the left ventricular endocardium for cardiac resynchronization therapy in non-responders and previously untreatable patients (SOLVE-CRT) trial. Am Heart J.

[13] Okabe T., Hummel J.D., Bank A.J. (2022). Leadless left ventricular stimulation with WiSE-CRT System – initial experience and results from phase I of SOLVE-CRT Study (nonrandomized, roll-in phase). Heart Rhythm.

[14] Glikson M., Nielsen J.C., Kronborg M.B. (2021). ESC Guidelines on cardiac pacing and cardiac resynchronization therapy. Eur Heart J.

[15] Huang W., Su L., Wu S. (2017). A novel pacing strategy with low and stable output: pacing the left bundle branch immediately beyond the conduction block. Can J Cardiol.

[16] Zhang S., Zhou X., Gold M.R. (2019). Left bundle branch pacing: JACC review topic of the week. J Am Coll Cardiol.

[17] Wang Y., Zhu H., Hou X. (2022). Randomized trial of left bundle branch vs biventricular pacing for cardiac resynchronization therapy. J Am Coll Cardiol.

[18] Diaz J.C., Sauer W.H., Duque M. (2023). Left bundle branch area pacing versus biventricular pacing as initial strategy for cardiac resynchronization. JACC Clin Electrophysiol.

[19] Elliott M.K., Jacon P., Sidhu B.S. (2021). Technical feasibility of leadless left bundle branch area pacing for cardiac resynchronization: a case series. Eur Heart J Case Rep.

[20] Sieniewicz B.J., Gould J.S., Rimington H.M., Ioannou N., Rinaldi C.A. (2017). Transseptal delivery of a leadless left ventricular endocardial pacing electrode. JACC Clin Electrophysiol.

[21] Yasmin F., Moeed A., Ochani R.K. (2024). Left bundle branch pacing vs biventricular pacing in heart failure patients with left bundle branch block: a systematic review and meta-analysis. World J Cardiol.

[22] Burri H., Jastrzebski M., Cano Ó. (2023). EHRA Clinical consensus statement on conduction system pacing implantation: endorsed by the Asia Pacific Heart Rhythm Society (APHRS), Canadian Heart Rhythm Society (CHRS), and Latin American Heart Rhythm Society (LAHRS). Europace.

[23] Jastrzębski M., Kiełbasa G., Cano O. (2022). Left bundle branch area pacing outcomes: the multicentre European MELOS study. Eur Heart J.

