# Supplementary material for: Feasibility of totally leadless left bundle branch area pacing via a left endocardial pacing electrode in patients with heart failure: A 3-case series
Source: HeartRhythm Case Rep. 2025 Aug 7;11(11):1120–5. doi: 10.1016/j.hrcr.2025.07.029 (PMC12666930; doi:10.1016/j.hrcr.2025.07.029)
Supplement: Supplemental Material [file mmc1.docx]

*Video : Fluoroscopic view showing the deployment of the WiSE-CRT endocardial pacing electrode on the left ventricular septum via a transseptal approach.*
